# Supplementary material for: Statins use and COVID-19 outcomes in hospitalized patients
Source: PLoS One. 2021 Sep 10;16(9):e0256899. doi: 10.1371/journal.pone.0256899 (PMC8432819; doi:10.1371/journal.pone.0256899)
Supplement: S2 Fig — (DOCX) [file pone.0256899.s002.docx]

**S2 Fig: Balance plot of raw and matched box and whisker plots of statin use versus statin non-use in severe COVID-19 infections**
