# Supplementary material for: Statins use and COVID-19 outcomes in hospitalized patients
Source: PLoS One. 2021 Sep 10;16(9):e0256899. doi: 10.1371/journal.pone.0256899 (PMC8432819; doi:10.1371/journal.pone.0256899)
Supplement: S1 Table — (DOCX) [file pone.0256899.s003.docx]

**S1 Table: Statin medications prescribed at the time of admission**

| **Generic Name** | **Number of Prescription-use** | **Percentage of Total** |
| --- | --- | --- |
| Atorvastatin | 444 | 74.75% |
| Pravastatin | 54 | 9.09% |
| Rosuvastatin | 54 | 9.09% |
| Simvastatin | 42 | 7.07% |
| **Total** | **594** | **100%** |
