# Supplementary material for: Statins use and COVID-19 outcomes in hospitalized patients
Source: PLoS One. 2021 Sep 10;16(9):e0256899. doi: 10.1371/journal.pone.0256899 (PMC8432819; doi:10.1371/journal.pone.0256899)
Supplement: S2 Table — (DOCX) [file pone.0256899.s004.docx]

**S2 Table: ACE inhibitors/ARBs prescribed at the time of admission**

| **Generic Name** | **Number of prescription use** | **Percentage of Total** |
| --- | --- | --- |
| Captopril | 35 | 8.64% |
| Enalapril | 4 | 0.99% |
| Enalaprilat | 1 | 0.25% |
| Lisinopril | 196 | 48.40% |
| Irbesartan | 8 | 1.98% |
| Losartan | 143 | 35.31% |
| Valsartan | 18 | 4.44% |
| **Total** | **405** | **100%** |
