# Supplementary material for: Statins use and COVID-19 outcomes in hospitalized patients
Source: PLoS One. 2021 Sep 10;16(9):e0256899. doi: 10.1371/journal.pone.0256899 (PMC8432819; doi:10.1371/journal.pone.0256899)
Supplement: S4 Table — (DOCX) [file pone.0256899.s006.docx]

**S4 Table: Means and variances in raw and balanced data for statin effect on severe COVID-19 disease**

|  | **Standardized Differences** | | **Variance Ratio** | |
| --- | --- | --- | --- | --- |
|  | **Raw** | **Matched** | **Raw** | **Matched** |
| **Age** | 1.215721 | -0.050198 | 0.641094 | 0.9921181 |
| **Sex** | 0.1619546 | 0.4294844 | 0.9808887 | 0.8237916 |
| **Race** | -0.4780291 | 0.0842316 | 0.8409711 | 0.9609955 |
| **Employment** | 0.8256554 | 0.134489 | 1.424941 | 0.827824 |
| **ARB/ACE – use** | 0.6711215 | 0.0354918 | 2.541085 | 1.073612 |
| **Hypertension** | 1.127974 | -0.0631645 | 0.7219009 | 0.972582 |
| **Diabetes** | 0.9781009 | -0.0270929 | 1.783245 | 0.9665398 |
| **Interval dialysis** | 0.3138432 | 0.0713279 | 6.281617 | 1.582475 |
